# Cognate base‐pair selectivity of hydrophobic unnatural bases in DNA ligation by T4 DNA ligase

**DOI:** 10.1002/bip.23407

**Published:** 2020-11-06

**Authors:** Michiko Kimoto, Si Hui Gabriella Soh, Hui Pen Tan, Itaru Okamoto, Ichiro Hirao

**Affiliations:** ^1^ Institute of Bioengineering and Nanotechnology A*STAR Singapore Singapore; ^2^ Raffles Institution Singapore Singapore

**Keywords:** genetic alphabet expansion, ligation, T4 DNA ligase, unnatural base pair

## Abstract

We present cognate base pair selectivity in template‐dependent ligation by T4 DNA ligase using a hydrophobic unnatural base pair (UBP), Ds‐Pa. T4 DNA ligase efficiently recognizes the Ds‐Pa pairing at the conjugation position, and Ds excludes the noncognate pairings with the natural bases. Our results indicate that the hydrophobic base pairing is allowed in enzymatic ligation with higher cognate base‐pair selectivity, relative to the hydrogen‐bond interactions between pairing bases. The efficient ligation using Ds‐Pa can be employed in recombinant DNA technology using genetic alphabet expansion, toward the creation of semi‐synthetic organisms containing UBPs.

## Graphical abstract

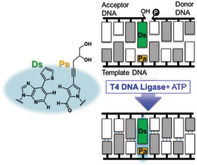

## 1 INTRODUCTION

The successful development of replicable unnatural base pairs (UBPs) with high fidelity has founded genetic alphabet expansion technologies.^[^ ^1^, ^2^, ^3^ ^]^ Practical applications *in vitro* and *in vivo* have rapidly advanced in the wide areas of novel quantitative PCR methods,^[^ ^4^, ^5^ ^]^ high‐affinity DNA aptamer generation,^[^ ^6^, ^7^ ^]^ RNA labeling,^[^ ^8^, ^9^ ^]^ and the creation of semi‐synthetic organisms for protein synthesis involving unnatural amino acids.^[^ ^10^, ^11^ ^]^ Basic DNA recombination technology involving UBPs expedites UBP‐applications. First, UB‐containing DNA (UB‐DNA) fragments are chemically synthesized using UB‐phosphoramidites. Second, the UB‐DNA fragments are ligated enzymatically to prepare long‐chain UBP‐DNAs. Third, these UB‐ or UBP‐DNAs are further amplified/replicated by PCR or inside cells by DNA polymerases. In this process, the enzymatic ligation involving UB/UBPs located close to the ligation sites remains enigmatic.^[^ ^12^, ^13^ ^]^

**FIGURE 1:**
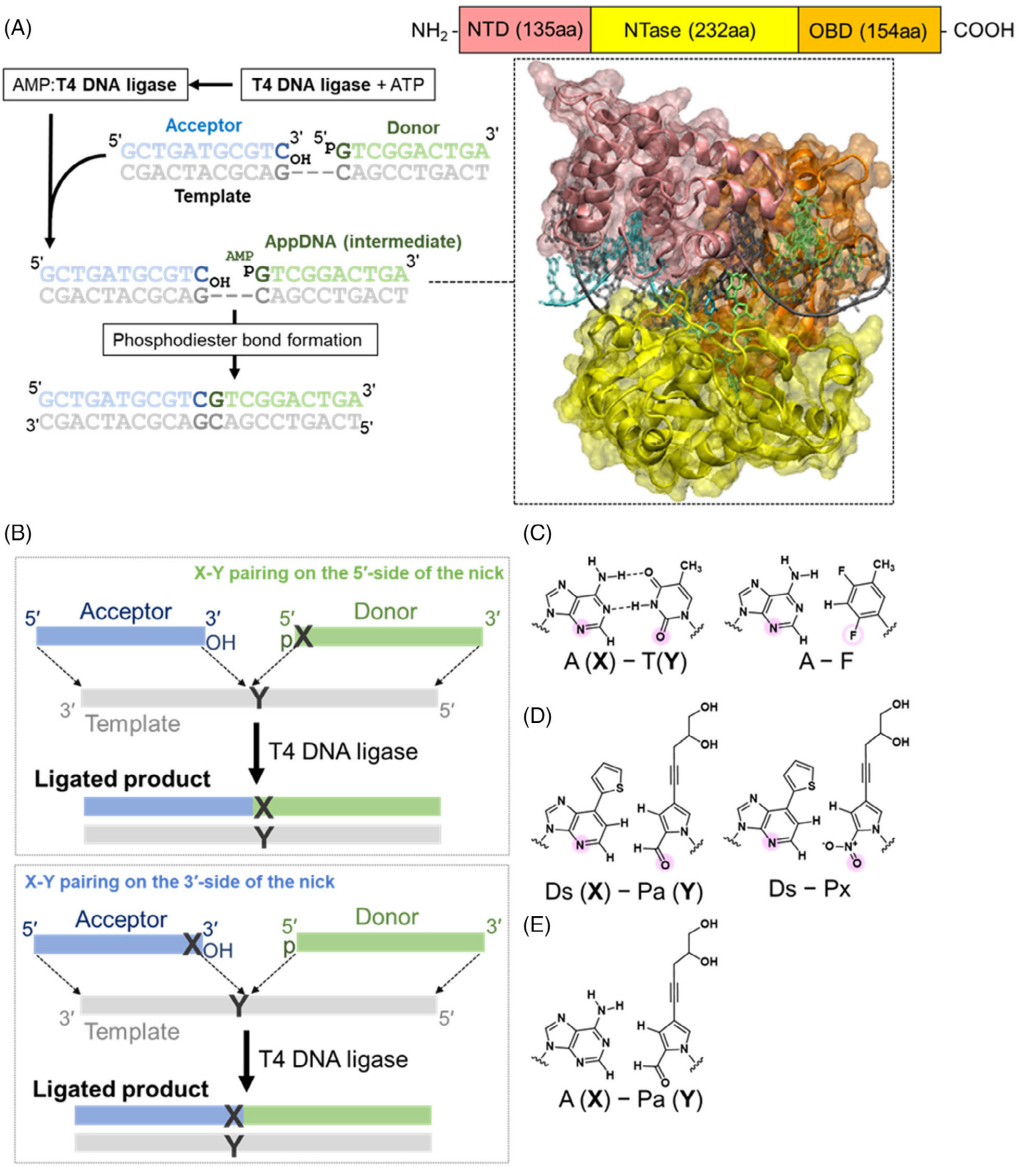
Enzymatic ligation by T4 DNA ligase using DNA with hydrophobic UBPs. A, T4 DNA ligase domain organization and the schematic representation of enzymatic ligation. The N‐terminal domain (NTD, pink) corresponds to the DNA‐binding domain (DBD). The nucleotidyl transferase domain (NTase, yellow) contains the catalytic core, and OBD (orange) is the oligonucleotide‐binding domain. The DNA duplex sequences and the complex structure of T4 DNA ligase with the DNA duplex, containing the adenylated DNA intermediate (AppDNA), were adopted from PDB: 6DT1. B, Enzymatic ligation reactions, where the UBP (X‐Y; e.g., Ds‐Pa) is located at the ligation junction (nick site). Chemical structures of A‐T and A‐F (C), Ds‐Pa and Ds‐Px (D), and A‐Pa (E). The minor groove hydrogen bond acceptor residues are indicated in solid circles (pink). The 2‐fluorine residue, which has lower hydrogen‐bond acceptor ability, is indicated in an open circle (pink)

T4 DNA ligase is a representative ATP‐dependent DNA ligase that is widely used for various *in vitro* and *in vivo* applications, and its ligation kinetics and mechanisms, including base‐pair selectivity, have been extensively studied (Figure 1).^[^ ^14^, ^15^, ^16^, ^17^, ^18^ ^]^ The N‐terminal domain (NTD) and DNA‐binding domain (DBD) of T4 DNA ligase bind to nicked double‐stranded DNA. The catalytic core of the nucleotidyl transferase domain (NTase) catalyzes the adenylation of the 5′‐phosphorylated donor strand with ATP, followed by the phosphodiester bond formation between the acceptor and adenylated donor strands (Figure 1A,B). The selectivity of the cognate natural base pairings is not high in T4 DNA ligation, and most of the noncognate mispairings (especially thymidine or guanosine in the template) at the ligation junction allow T4 DNA ligase to mediate the ligation.^[^ ^15^, ^19^ ^]^ However, as also found in the *Escherichia coli* and *Thermus thermophilus* DNA ligases, T4 DNA ligase exhibits greater discrimination against the mismatches on the 3′‐side of the nick in comparison with those on the 5′‐side of the nick (Figure 1B).^[^ ^16^, ^17^, ^20^, ^21^, ^22^ ^]^

Many reports have described the T4 DNA ligase activity in DNA ligation involving modified‐base and UB nucleotides.^[^ ^12^, ^13^, ^23^, ^24^, ^25^ ^]^ The studies revealed several trends in the T4 DNA ligase activity. (1) T4 DNA ligase is more tolerant of modified bases at the 5′‐end of the phosphate donor strand than those at the 3′‐end of the acceptor strand,^[^ ^23^, ^24^ ^]^ which are similar tendencies to those found in the mismatch base pairing recognitions.^[^ ^17^ ^]^ (2) Modified bases in the template strand are more sensitive than those in the acceptor or donor strands.^[^ ^23^, ^24^ ^]^ (3) Modifications on the major groove side are more acceptable, as compared to those on the minor groove side.^[^ ^25^ ^]^ (4) Minor groove hydrogen bond acceptor residues in UBPs are important, as also found in replication by DNA polymerases.^[^ ^23^, ^26^ ^]^ One of the UBs, F, is an isostere of T but has the 2‐fluorine residue,^[^ ^27^, ^28^ ^]^ which has lower hydrogen‐bond acceptor ability than that of the keto group at position 2 of pyrimidines, and thus the A‐F pair is less effective than the A‐T pair in phosphodiester bond formation by T4 DNA ligase (Figure 1C).^[^ ^23^ ^]^ (5) Hydrogen‐bonded UBPs, such as isoG‐isoC, are well tolerated.^[^ ^12^ ^]^ (6) In the presence of 20% (v/v) DMSO and a large excess of T4 DNA ligase, the phosphodiester bond formation occurs efficiently with either of the two hydrophobic UBs, 5SICS and NaM, at the 3′‐end of the acceptor strand, with any natural bases on the template (mostly efficient ligation was with G).^[^ ^13^ ^]^ Nonetheless, no studies have investigated the ligation efficiencies and selectivities of the nonhydrogen‐bonded cognate UBPs that function in replication as a third base pair with high fidelity.

In this paper, we explored the phosphodiester bond formation by T4 DNA ligase with one of our nonhydrogen‐bonded hydrophobic UBPs, Ds‐Pa (Figure 1D),^[^ ^29^ ^]^ which was designed as a UBP according to the concept of differing shape complementarity from the natural base pairs. Another UBP, Ds‐Px (Figure 1D),^[^ ^30^, ^31^, ^32^ ^]^ was also developed as a third base pair with higher fidelity than the Ds‐Pa pair, since Pa also pairs with A to some extent in replication (Figure 1E).^[^ ^29^ ^]^ However, the stability of the Px nucleoside is relatively low under the basic conditions in DNA chemical synthesis. Thus, in this study, we used the Ds‐Pa pair: Ds‐containing DNAs for the acceptor and the donor strands, and Pa‐containing DNAs for the template strands.

We expected that the Ds‐Pa pair would not disturb the double‐stranded helix structure at the ligation junction. Although we have no direct structural data of the Ds‐Pa pair in DNA, other analyses using UBPs related to Ds‐Pa indicated the similar geometry of the Ds‐Pa pair to those of the natural base pairings within the active site of T4 DNA ligase (Figure S1). The NMR structural analysis of the Q‐Pa pair,^[^ ^33^ ^]^ in which the Q base has a methyl group at the position of the thienyl group of the Ds base and was originally developed as an A isostere,^[^ ^34^ ^]^ revealed the structural resemblance between Q‐Pa and A‐T. The X‐ray crystallography of the Ds‐Px pair in a ternary complex of DNA polymerase, Ds‐template and primer duplex, and the substrate of Px verified our expected structure, as indicated in Figure 1D.^[^ ^35^ ^]^

Surprisingly, the Ds‐Pa pair exhibited high selectivity in T4 DNA ligation when the Ds base is located at the ligation junction of either an acceptor strand or a donor strand, as compared to the natural base pairs. The ligation efficiency involving the Ds‐Pa pair was similar to that of natural‐base DNA duplexes. Our analyses of the selective discrimination of the Ds‐Pa pair demonstrate the high availability of the hydrophobic UPBs as the base pair at the ligation junction in T4 DNA ligation.

## 2 MATERIALS AND METHODS

### 2.1 Materials

The unnatural dDs substrate (dDsTP) was synthesized as described previously.^[^ ^29^ ^]^ Phosphoramidites of unnatural bases, dDs and dPa (benzoyl group protection for two hydroxyl groups of the diol side chain), were chemically synthesized in‐house. DNA fragments containing unnatural dDs and dPa were chemically synthesized with a DNA synthesizer, Applied Biosystems 392 DNA synthesizer or H‐8‐SE (K&A Laborgeraete), using the Ds‐, Pa‐, and natural‐base‐amidites (Glen Research). Natural‐base DNA fragments (including FAM‐labeled at the 5′‐end) were purchased from Integrated DNA Technologies, or chemically synthesized in‐house. DNA fragments were purified on denaturing polyacrylamide gels. The sequences of the fragments used in this study are summarized in Table S1. Klenow fragment 3′ → 5′ exo‐ (KF exo‐), T4 polynucleotide kinase (PNK), 10× T4 PNK reaction buffer and 10× ligation reaction buffer were from New England Biolabs (NEB). SYBR Gold, and T4 DNA ligase were purchased from Thermo Fisher Scientific. Urea and 10× TBE were purchased from first BASE. The 40% acrylamide solution (19:1) was purchased from Bio‐Rad Laboratories.

### 2.2 Chemical synthesis of Pa‐amidite

#### 2.2.1 General information for chemical synthesis

All reagents and solvents were purchased from standard suppliers (Tokyo Chemical Industry Co., Ltd., Sigma‐Aldrich, and Merck). Thin layer chromatography was performed using TLC silica gel 60 F254 plates (Merck). Compounds were visualized by UV shadowing or staining with a sulfuric acid‐methanol solution. Nucleoside derivatives were purified on a Gilson HPLC system with a preparative C18 column (μ‐BONDASPHERE, Waters, 19 mm × 150 mm). ^1^H NMR and ^31^P NMR spectra were recorded on a Bruker magnetic resonance spectrometer. CDCl_3_ and DMSO‐d_6_ were used as the solvents.

#### 2.2.2 (S)‐Pent‐4‐yne‐1,2‐diyl dibenzoate (2)

Lithium acetylide ethylenediamine complex (8.31 g, 81.2 mmol) was dissolved in hexamethylphosphoric triamide (20 mL) and dry THF (80 mL), and the resulting mixture was cooled to 0 °C. Afterward, (R)‐(+)‐glycidol, compound **1**, (1786 μL, 27 mmol) in dry THF (40 mL) was added dropwise with stirring at 0 °C. The reaction mixture was stirred for 15.5 hours at ambient temperature, and then saturated NH_4_Cl (200 mL) was added. The mixture was extracted with EtOAc (50 mL × 3). The combined organic phase was dried over MgSO_4_ and concentrated under reduced pressure. The residue was co‐evaporated with dry pyridine twice. Benzoyl chloride (12.5 mL, 108 mmol) was added to the residue in dry pyridine (60 mL). The resulting mixture was stirred for 19 hours at ambient temperature. The reaction was quenched by the addition of methanol (10 mL) and stirred for 30 minutes at ambient temperature, prior to concentration under reduced pressure. EtOAc (150 mL) and water (150 mL) were poured into the resulting residue. The organic layer was separated and washed with water (150 mL), saturated aq‐NaHCO_3_ (150 mL), and brine (150 mL). The organic phase was dried over MgSO_4_ and concentrated under reduced pressure. The resulting residue was purified by silica gel column chromatography (150 g of silica gel, hexane/EtOAc = 100:0 to 95:5) to give compound **2** (2.86 g, 9.26 mmol, 34%). ^1^H NMR (400 MHz, CDCl_3_, *δ*, ppm) 8.08‐8.02 (m, 4H), 7.60‐7.54 (m, 2H), 7.47‐7.41 (m, 4H), 5.58‐5.53 (m, 1H), 4.68 (dq, 2H, *J*_1_ = 12.0 Hz and *J*_2_ = 3.9 Hz), 2.80 (dd, 2H, *J*_1_ = 6.2 Hz and *J*_2_ = 2.6 Hz), 2.08 (t, 1H, *J* = 2.6 Hz).

#### 2.2.3 1‐(2‐Deoxy‐β‐D‐ribofuranosyl)‐(S)‐4‐(4,5‐dibenzoyloxy‐pent‐1‐yn‐1‐yl)‐1H‐pyrrole‐2‐carbaldehyde (3)

A mixture of iodo‐dPa (1.94 g, 5.75 mmol), copper iodide (175 mg, 0.92 mmol), tetrakis (triphenylphosphine)palladium(0) (332 mg, 0.288 mmol), triethylamine (1.6 mL, 11.5 mmol), and DMF (30 mL) was stirred and degassed for 10 minutes under reduced pressure, and then flushed with argon. To this mixture was added compound **2** (2.22 g, 7.19 mmol), and the resulting mixture was further degassed for 10 minutes under reduced pressure and flushed with argon, prior to stirring for 4 hours at ambient temperature. The reaction mixture was concentrated under reduced pressure. The resulting dark liquid mixture was purified by silica gel column chromatography (60 g of silica gel, DCM/methanol =100:0 to 98:2) and C18 RP‐HPLC (eluted by a gradient of CH_3_CN [40%‐80%] in H_2_O) to give compound **3** (2.35 g, 4.53 mmol, 79%). ^1^H NMR (400 MHz, DMSO‐d_6_, δ, ppm) 9.47 (d, 1H, *J* = 0.9 Hz), 8.00‐7.95 (m, 4H), 7.87 (s, 1H), 7.70‐7.64 (m, 2H), 7.56‐7.50 (m, 4H), 7.08 (d, 1H, *J* = 1.8 Hz), 6.66 (t, 1H, *J* = 6.3 Hz), 5.57‐5.52 (m, 1H), 5.27 (d, 1H, *J* = 4.1 Hz), 5.03 (t, 1H, *J* = 5.3 Hz), 4.74‐4.70 (dd, 1H, *J*_1_ = 11.9 Hz and *J*_2_ = 3.3 Hz), 4.64‐4.59 (dd, 1H, *J*_1_ = 12.0 Hz and *J*_2_ = 6.7 Hz), 4.24 (m, 1H), 3.81 (dt, 1H, *J*_1_ = 4.0 Hz and *J*_2_ = 3.6 Hz), 3.62‐3.50 (m, 2H), 3.03 (d, 2H, *J* = 6.4 Hz), 2.32‐2.08 (m, 2H).

#### 2.2.4 1‐(5‐O‐DMTr‐2‐deoxy‐β‐D‐ribofuranosyl)‐(S)‐4‐(4,5‐dibenzoyloxy‐pent‐1‐yn‐1‐yl)‐1H‐pyrrole‐2‐carbaldehyde (4)

Compound **3** (2.35 g, 4.53 mmol) was co‐evaporated with dry pyridine three times. The residue in dry pyridine (40 mL) was mixed with 4,4′‐dimethoxytrityl chloride (DMTrCl, 1.84 g, 5.44 mmol). The resulting mixture was stirred for 2 hours at ambient temperature, prior to concentration under reduced pressure. EtOAc (150 mL) and water (150 mL) were poured into the resulting residue. The organic layer was separated and washed with saturated aq‐NaHCO_3_ (150 mL × 1) and brine (150 mL × 1). After drying with MgSO_4_, the solvent was evaporated under reduced pressure. The residue was purified by silica gel column chromatography (60 g of silica gel, hexane/EtOAc = 100:0 to 70:30) to give compound **4** (2.98 g, 3.63 mmol, 80%). ^1^H NMR (400 MHz, DMSO‐d_6_, *δ*, ppm) 9.47 (d, 1H, *J* = 0.8 Hz), 7.98‐7.94 (m, 4H), 7.68‐7.63 (m, 3H), 7.57‐7.47 (m, 4H), 7.39‐7.37 (m, 2H), 7.31‐7.19 (m 6H), 7.11 (d, 1H, *J* = 1.8 Hz), 6.89‐6.87 (m, 4H), 6.67 (t, 1H, *J* = 5.9 Hz), 5.54‐5.49 (m, 1H), 5.36 (d, 1H, *J* = 3.8 Hz), 4.71‐4.67 (dd, 1H, *J*_1_ = 11.9 Hz and *J*_2_ = 3.3 Hz), 4.60‐4.55 (dd, 1H, *J*_1_ = 12.0 Hz and *J*_2_ = 6.7 Hz), 4.26 (m, 1H), 3.97‐3.93 (m, 1H), 3.73 (d, 6H, *J* = 1.0 Hz), 3.22‐3.18 (dd, 1H, *J*_1_ = 10.4 Hz and *J*_2_ = 5.8 Hz), 3.14‐3.11 (dd, 1H, *J*_1_ = 10.4 Hz and *J*_2_ = 3.1 Hz), 2.99 (d, 2H, *J* = 6.4 Hz), 2.36‐2.18 (m, 2H).

#### 2.2.5 1‐(5‐O‐DMTr‐2‐deoxy‐β‐D‐ribofuranosyl)‐(S)‐4‐(4,5‐dibenzoyloxy‐pent‐1‐yn‐1‐yl)‐1H‐pyrrole‐2‐carbaldehyde phosphoramidite (5)

Compound **4** (2.98 g, 3.63 mmol) was co‐evaporated with pyridine three times and then with dry THF three times. *N,N*‐Diisopropylethylamine (950 μL, 5.45 mmol) and 2‐cyanoethyl *N,N*‐diisopropylchlorophosphoramidite (893 μL, 4 mmol) were added to the residue in anhydrous THF (35 mL), and the resulting mixture was stirred for 3 hours at ambient temperature. Dry methanol (500 μL) was added to the mixture to quench the reaction. EtOAc/triethylamine (150 mL, 99/1) and saturated aq‐NaHCO_3_ (150 mL) were poured into the resulting residue. The organic layer was separated and washed with aq‐NaHCO_3_ (150 mL) and brine (150 mL). After drying with MgSO_4_, the solvent was evaporated under reduced pressure. The residue was purified by silica gel column chromatography (80 g, hexane/EtOAc = 100/0 to 80/20 containing 1% triethylamine) to give compound **5** (2.84 g, 2.78 mmol, 76%). ^1^H NMR (400 MHz, DMSO‐d_6_, *δ*, ppm) 9.52‐9.50 (m, 1H), 7.97‐7.94 (m, 4H), 7.69‐7.62 (m, 3H), 7.52‐7.47 (m, 4H), 7.40‐7.36 (m, 2H), 7.31‐7.18 (m, 6H), 7.12 (m, 1H), 6.89‐6.86 (m, 4H), 6.74‐6.68 (m, 1H), 5.55‐5.48 (m, 1H), 4.71‐4.46 (m, 3H), 4.12‐4.04 (m, 1H), 3.73‐3.72 (m, 6H), 3.67‐3.46 (m, 3H), 3.27‐3.17 (m, 2H), 2.99 (t, 2H, *J* = 5.9 Hz), 2.76 (t, 1H, *J* = 5.9 Hz), 2.66 (t, 1H, *J* = 5.9 Hz), 2.49‐2.32 (m, 2H), 1.14‐0.99 (m, 12H). ^31^P NMR (162 MHz, DMSO‐d_6_, *δ*, ppm) 147.8 and 147.5 (diastereoisomers).

### 2.3 Phosphorylation of the 5′‐end of DNA fragments used as the donor strands

The 5′‐end of each DNA fragment (6.25 μM) used as the donor strand was phosphorylated, using 1 mM ATP and 0.2 U/μL T4 PNK in 1× T4 PNK reaction buffer (70 mM Tris‐HCl, pH 7.6, 10 mM MgCl_2_, and 5 mM DTT). After a 30‐minutes incubation, the T4 PNK was inactivated by heating at 65 °C for 20 minutes. The solution was directly used for the subsequent ligation, without further purification.

### 2.4 dsDNA ligation by T4 DNA ligase

Each set of the acceptor strand (5 pmol, without FAM‐labeling at the 5′‐end), the donor strand (5 pmol), and the corresponding template strand (5 pmol) was mixed, and then annealed by heating the DNA solution (5 μL) at 95 °C for 3 minutes, followed by slow cooling to 4 °C at a rate of 0.1 °C/sec. The dsDNA solution was mixed with 5 μL of reaction solution, containing T4 DNA ligase (2.5 Weiss units) in 2× ligation reaction buffer (100 mM Tris‐HCl, pH 7.6, 20 mM MgCl_2_, 2 mM ATP, and 20 mM DTT). The final concentrations of each DNA fragment and T4 DNA ligase were 500 nM and 0.25 Weiss U/μL, respectively. The reaction was performed at 22 °C for 10 minutes, and stopped by adding 10 μL of 10 M urea/1× TBE containing 0.025% (w/v) bromophenol blue (the stopping solution), immediately followed by heating at 75 °C for 3 minutes. Portions of the ligation products (10 μL) were then loaded on a 20% denaturing polyacrylamide gel containing 7 M urea, and fractionated by electrophoresis. The DNA band patterns on the gels were detected with an LAS‐4000 bio‐imager (Fuji Film), after staining with SYBR Gold.

To quantify the yield of each ligation reaction at different reaction time points, acceptor strand with FAM‐labeling at the 5′‐end were used for fluorescence detection, in place of SYBR Gold staining. Each set of the labeled acceptor strand, the donor strand and the corresponding template strand was mixed in 1× ligation reaction buffer at 677 nM each, and then annealed. The annealed dsDNA solution (7.5 μL) was mixed with 2.5 μL of T4 DNA ligase solution (0.5 or 0.025 Weiss units) in 1× ligation reaction buffer to obtain final concentrations of 500 nM (each DNA fragment) and 0.05 or 0.0025 Weiss U/μL (T4 DNA ligase), respectively. The reaction was performed at 22 °C and stopped at various time points from 1 to 22 minutes, by adding 10 μL of the stopping solution, followed by immediately heating at 75 °C for 3 minutes. Portions of the ligated products (8 μL) were then analyzed by denaturing gel electrophoresis. The FAM‐labeled DNA bands on the gel were detected and quantified with the LAS‐4000 bio‐imager (SYBR mode). The yield [Y (%), the percentage of the ligated 40‐mer products/(the ligated 40‐mer and nonligated acceptor strand)] was calculated for each time point and plotted over each time point, *t* (min). To estimate the turnover numbers of the ligation, we used the yield at 5 minutes, since the fitting of a line to the early part of the curve (up to 5 minutes) is well correlated with the yield, thus allowing the detection of experimental errors. The original concentration of T4 DNA ligase used (5 Weiss U/μL) used in this study was estimated as 0.4 ± 0.1 mg/mL (7.2 ± 1.8 μM using molecular weight of 55.3 kDa), based on a Pierce 660 protein assay (Standard: BSA), silver staining of the band in SDS‐PAGE gel (Standard: BSA), and absorbance at 280 nm after buffer exchange (removing 50% glycerol, *ε*_280_ = 57 675 M^−1^ cm^−1^). Thus, a 10% yield (500 nM dsDNA, 0.0025 Weiss U/μL) for 5 minutes corresponds to a 0.0463 turnover number (*k*_obs_ [seconds^−1^], the estimated error would be ±25%).^[^ ^18^ ^]^

## 3 RESULTS AND DISCUSSIONS

### 3.1 Chemical synthesis of the Pa‐amidite derivative for DNA chemical synthesis

Since the Pa base is modifiable with any functional groups as a side chain, we chose diol‐modified Pa for the experiments. This is because the diol modification of the Px moiety provides good fitting in DNA polymerase recognition^[^ ^35^ ^]^ and allows high PCR‐amplification fidelity.^[^ ^31^, ^32^ ^]^ We chemically synthesized the Pa‐amidite, in which the diol moiety was protected with benzoyl groups (Scheme S1). At first, we used acetyl groups for the diol residue protection. However, the acetyl groups were not stable under mildly basic conditions, and thus we chose the benzoyl groups for the protection. The benzoyl‐protected pent‐4‐yne‐1,2‐diol (**2**) was derived from (*R*)(+)‐glycidol (**1**). The iodo‐Pa derivative was coupled with compound **2**, followed by dimethoxytritylation and phosphoramidation, to yield the benzoyl‐protected Pa‐amidite. These compounds were characterized by ^1^H‐ and ^31^P‐NMR (Figures S2 and S3). We confirmed the high stability of the deprotected Pa‐nucleoside, and no decomposition was observed after a treatment with concentrated ammonia at 55 °C for 6 hours (Figure S4). Using the Pa‐amidite, we synthesized Pa‐containing DNA fragments by a conventional method (Figure S5; ESI‐MS data, Table S1). We also prepared Ds‐containing DNA fragments by chemical synthesis, using the Ds‐amidite^[^ ^29^ ^]^ in combination with enzymatic insertion (Figure S6).

### 3.2 Ligation efficiency and selectivity of Ds at the 5′‐ends of the ligation junction in the donor strand

**FIGURE 2:**
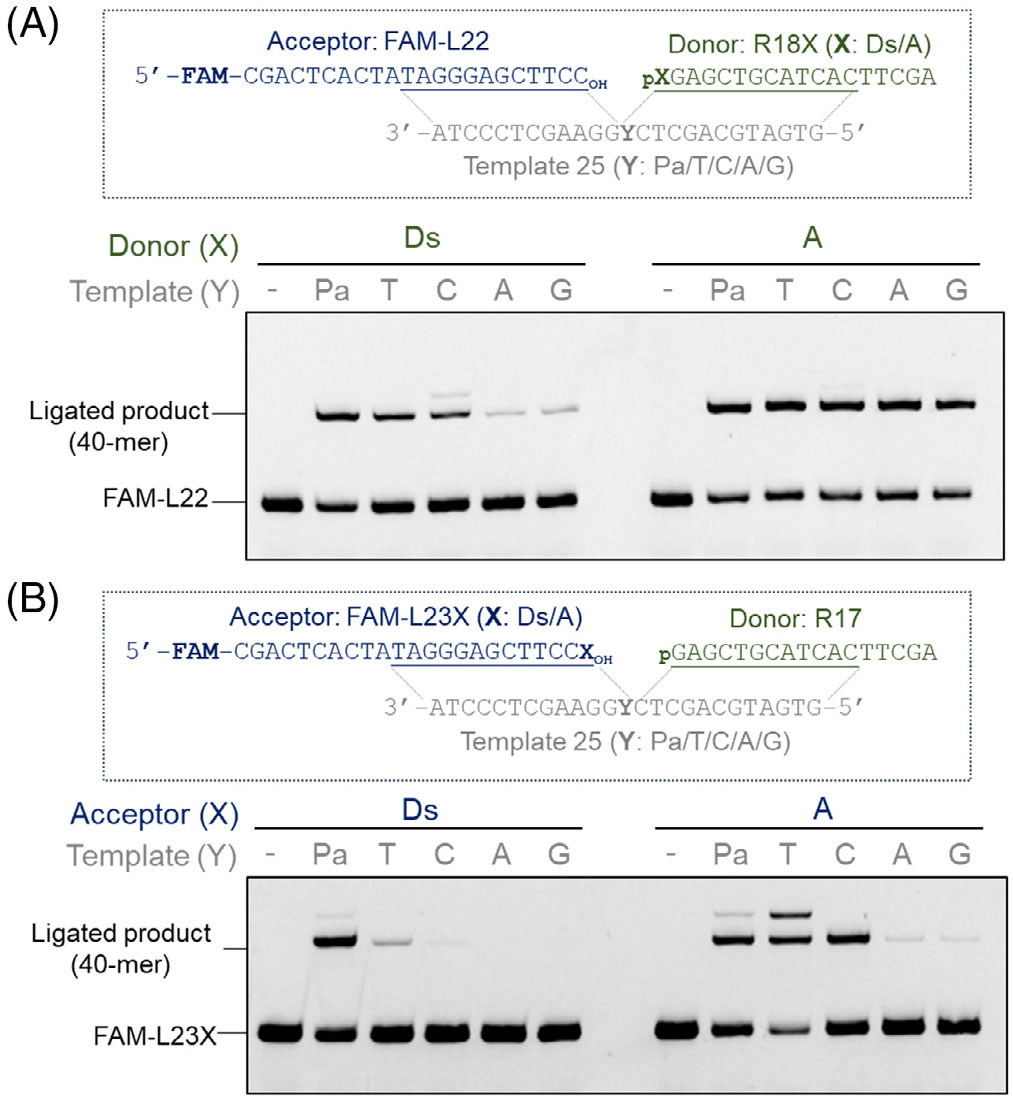
Ligation of (A) 5′‐phosphorylated R18X (donor strand, X = Ds or A) to FAM‐L22 (acceptor strand) and (B) 5′‐ phosphorylated R17 (donor strand) to FAM‐L23X (acceptor strand, X = Ds or A) in the presence of Template 25 (Y = Pa, T, C, A, or G). Reaction conditions: 0.05 Weiss U/μL T4 DNA ligase (corresponding to 1/5 of the amounts of ligase used in Figures 2 and 3), 0.5 μM each DNA, 10 minutes at 22 °C. The FAM‐labeled DNA bands in the gel were detected with an LAS4000 bio‐imager. The ligated products are 40‐mers. The DNA species higher than 40‐mer are expected to be nontemplate ligated products (with one extra donor strand, since we did not block the 3′‐end)

First, we examined the ligation of Ds at the 5′‐end of the ligation junction, using an 18‐mer donor DNA fragment, R18Ds. We also prepared R18A, containing A instead of Ds in R18Ds, as a control. Prior to the ligation experiments, the 5′‐termini of R18Ds and R18A were phosphorylated by T4 polynucleotide kinase. Each of these 5′‐phosphorylated DNA fragments, R18X (X = Ds or A), and a 22‐mer acceptor strand, L22, were annealed with a 25‐mer template strand, Template 25 (Y = Pa, T, C, A or G) (Figure 2A and Figure S7). After an incubation with T4 DNA ligase and ATP at 22 °C for 10 minutes, the ligation products (40‐mer) were analyzed on a denaturing gel.

The highly efficient ligation of R18Ds with L22 was observed in the Ds‐Pa pairing, and the ligation efficiencies (Ds‐Pa > Ds‐pyrimidine > Ds‐purine pairings) were correlated with the shape complementarities among the cognate and noncognate pairings, as also shown in the replication tendencies by DNA polymerases.^[^ ^29^ ^]^ In contrast, R18A was efficiently ligated with the acceptor strand in all of the cognate A‐T and noncognate pairings, including the A‐Pa pairing, after 10 minutes. The ligation efficiency involving the Ds‐Pa pair was as high as that involving the A‐T pair. Furthermore, under these conditions, the selectivity of the Ds‐Pa pair was higher than that of the natural base pairs: In contrast to A, Ds efficiently eliminated noncognate pairing with A or G in ligation.

These single‐time‐point results suggest that the nonhydrogen‐bonded Ds‐Pa pair allows T4 DNA ligase to mediate the ligation reaction involving the UBP at the 5′‐ends of the ligation junction (the 5′‐side of the nick), through a certain discrimination of Ds. However, the ligation reactions with A at the 5′‐end in the donor strand, using any template with Pa, T, C, A, and G, are very fast and completed in much less than 10 minutes, which might mask any differences in the rates with cognate against noncognate base pairs. In contrast, the ligation reactions with Ds at the 5′‐end in the donor strand might be much slower, allowing the detection of apparent differences in rates. Thus, we next analyzed the time course of the ligation reactions for a careful comparison of the rates of ligations.

**FIGURE 3:**
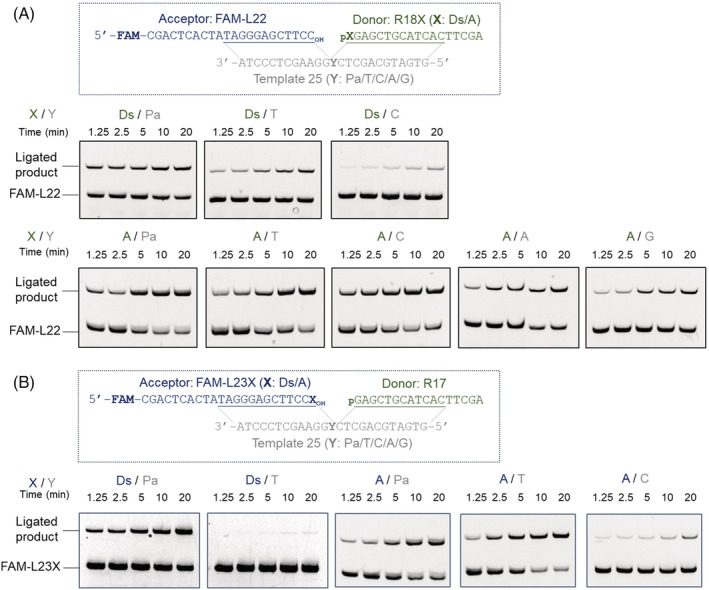
Time course of the ligation reactions. A, Ligation of 5′‐phosphorylated R18X (donor strand) to FAM‐L22 (acceptor strand) in the presence of Template 25. B, Ligation of 5′‐phosphorylated R17 (donor strand) to FAM‐L23X (acceptor strand) in the presence of Template 25. X = Ds or A; Y = Pa, T, C, A, or G. Reaction conditions: 0.0025 Weiss U/μL T4 DNA ligase, 0.5 μM of each DNA at 22 °C. The time course of the reactions with the various base pairs were assessed by performing the ligation reactions at various time intervals: 1.25, 2.5, 5, 10 and 20 minutes

**FIGURE 4:**
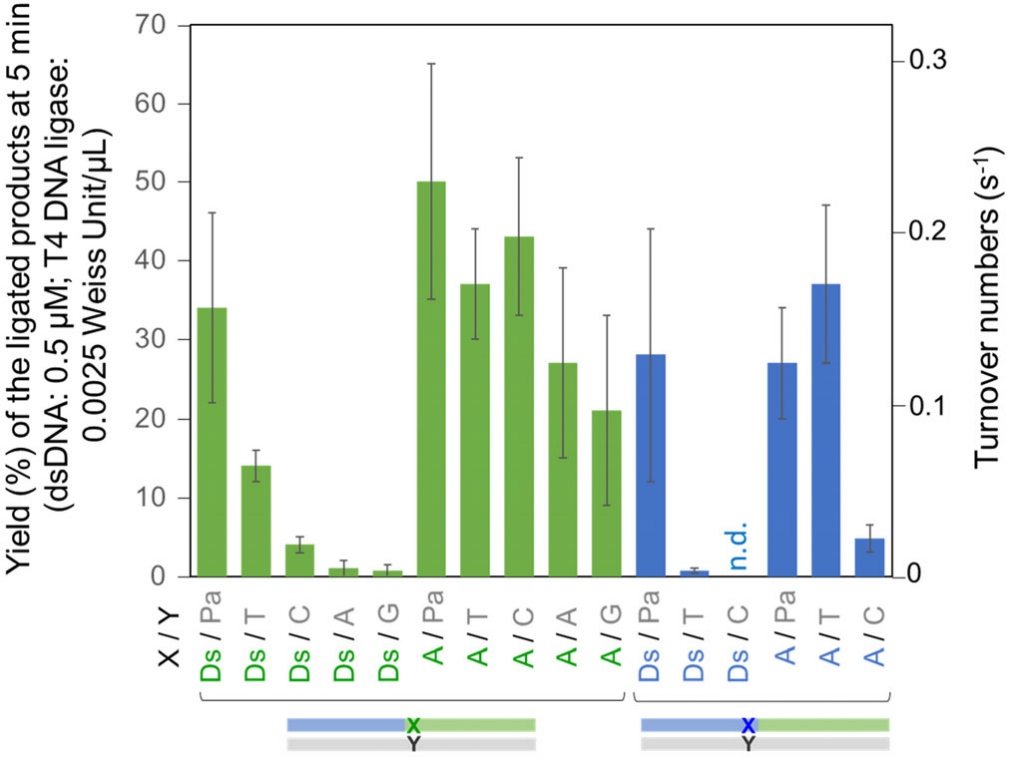
Summary of ligation efficiencies with the hydrophobic unnatural Ds base at the 5′‐end of the donor strand or at the 3′‐end of the acceptor strands. The rates of the ligation (turnover numbers) were calculated from the yields of the ligated products at 5 minutes (dsDNA: 0.5 μM; T4 DNA ligase: 0.0025 Weiss U/μL, 3.6 ± 0.9 nM) by averaging independent repetitive data (n = 2‐4, Figures 3, S8 and S10), and indicated with the standard deviations

For the time course reactions, we used FAM‐labeled acceptor strands to simplify the quantification of the ligated product yields (Figure 3A), instead of DNA detection with SYBR‐Gold staining (Figure S7), and reduced the amount of the T4 DNA ligase to as low as 1/100. From the yields of the ligated products at various time points (1‐22 minutes) (Figure S8), we estimated the rates of ligations (*k*_obs_: turnover numbers, Figure 4) at 500 nM concentrations of dsDNA, which showed the steady state turnover for a linear rate. Using this method, we determined that the turnover numbers for the A‐T pair were 0.17 ± 0.03 (seconds^−1^), which is very close to the value of 0.13 ± 0.04 (seconds^−1^) in the previous report using similar sequence contexts.^[^ ^18^ ^]^ In the case of the ligation reactions with A at the 5′‐end in the donor strand, the order for the rates was A‐Pa ≈ A‐T ≈ A‐C ≥ A‐A ≥ A‐G, thus clarifying the small differences in ligation turnover between A‐pyrimidines/Pa and A‐purines (Figure 4). However, there were still no clear differences among the A pairings with Pa, T, and C, as compared to the cases of the Ds pairings (Figure 4). Thus, we confirmed that the ligated product band patterns in Figure 2A and Figure S7 reflected the higher selectivity of the Ds‐Pa pair in ligation.

These results also indicated the similar minor groove interaction pattern in the Pa base moiety, found between the proton acceptor residues of natural and unnatural bases with T4 DNA ligase. Initially, we derived the Pa base from difluorotoluene (F) to improve the shape complementarity with its pairing bases, such as Ds‐Pa and A‐Pa, by utilizing the five‐membered ring.^[^ ^33^ ^]^ In addition, the oxygen of the aldehyde group of Pa increases the interaction with DNA polymerases.^[^ ^33^ ^]^ In contrast, the hydrogen‐bond acceptor ability of the fluorine in the F base is 10‐fold lower than that of the 2‐keto group of pyrimidine.^[^ ^36^ ^]^

The bulky shape of Ds, which is larger than those of A and G, effectively excludes the noncognate pairings with the purine bases and partially impedes those with the pyrimidine bases. One possible reason for this highly efficient exclusion of the Ds pairing with A and G, as compared to that of the A pairing with the purines, is the tight stacking structure of the Ds base with the neighboring bases in the duplex DNA, which reduces the flexibility for the Ds‐purine pairings. In the ligations involving the noncognate pairing with Ds, the adenylated intermediate fragments of R18Ds (App) were also observed (Figure S7). Thus, the adenylation by T4 DNA ligase tolerates the different geometries of the noncognate pairings with Ds, but the phosphodiester bond formation process by T4 DNA ligase is more sensitive to the lower shape complementarity of the noncognate pairs with Ds.

### 3.3 Ligation efficiency and selectivity of Ds at the 3′‐ends of the ligation junction in the acceptor strand

Next, we examined the ligation of Ds at the 3′‐end of the ligation junction, using L23Ds as the acceptor strand and L23A as the control (Figures 2B, 3B, and S9). The L23Ds fragment containing Ds at the 3′‐terminus was prepared by single‐nucleotide insertion, using dDsTP and the complementary strand containing Pa with exonuclease‐deficient Klenow fragment (Figure S6). The acceptor strand, L23X (X = Ds or A), and the 17‐mer or 57‐mer donor strand, R17 (Figures 2B and 3B) or R57 (Figure S9), were annealed with the 25‐mer template strands, prior to the ligation reaction.

The ligation selectivity of the Ds‐Pa pair at the acceptor position (L23Ds in Figures 2B and 3B) was much higher than that at the donor position (R18Ds in Figures 2A, 3A, and 4). Similarly, the ligation selectivity of the A‐T pair at the acceptor position was also higher than that at the donor position. The differences of the ligation efficiencies observed by the analysis of a single time point (Figure 2B) were well correlated with the those analyzed through the time course of the ligation reactions (Figures 3B, 4, and S10). These results are well consistent with previous reports that T4 DNA ligase is less tolerant of the noncognate pairings involving modified nucleotides at the 3′‐end of the acceptor strands, as compared to those at the 5′‐terminus of the donor strands.^[^ ^23^, ^24^ ^]^ Under these circumstances, the selectivity of the Ds‐Pa pair in T4 DNA ligase recognition was higher than that of the A‐T pair (Figure 4).

### 3.4 Ligation using four strands involving UBs

**FIGURE 5:**
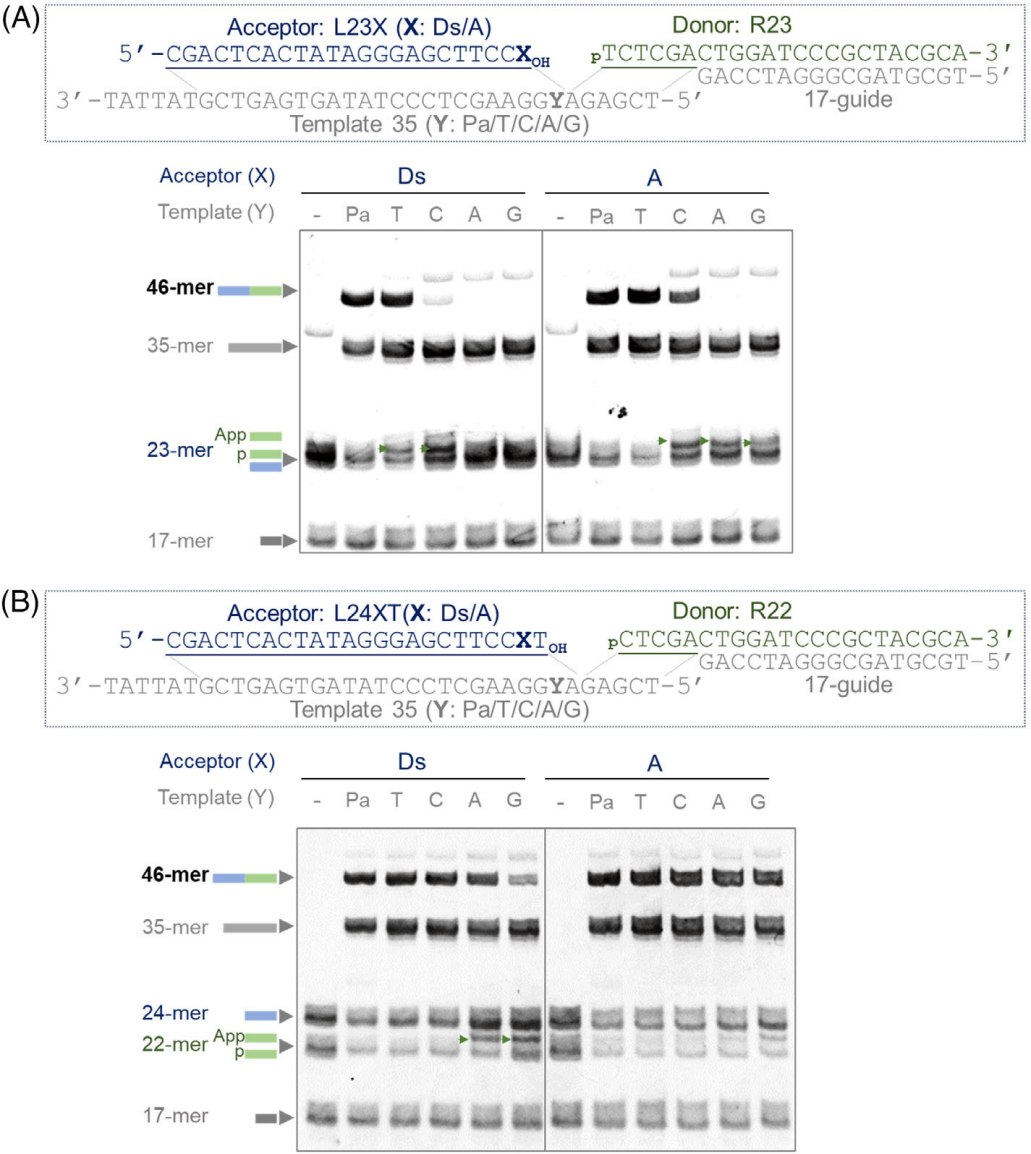
Ligation of 5′‐phosphorylated R23 (A) or R22 (B) (donor strand) to L23X (A) or L24XT (B) (acceptor strand) in the presence of Template 35 (template strand). A 17‐mer DNA (17‐guide) was added to ensure the sufficient length of the duplex formation for T4 DNA ligase recognition. Reaction conditions: 0.25 Weiss U/μL T4 DNA ligase, 0.5 μM of each DNA fragment, 10 minutes at 22 °C. X = Ds or A, Y = Pa, T, C, A, or G. The DNA species higher than 46‐mer and those between 35‐mer and 46‐mer are expected to be nontemplate ligated products (with one extra donor strand, since we did not block the 3′ end)

To demonstrate the practical ligation of double‐stranded DNAs, we performed a sticky‐end type ligation using four DNA strands, in which only the R23 strand was 5′‐phosphorylated, and thus only the upper strands were ligated (Figure 5A). We confirmed the highly efficient sticky‐end type ligation involving the Ds‐Pa pairing (Figure 5A). The Ds‐containing acceptor strand, L23Ds, was ligated with R23 in the Ds‐Pa and Ds‐T pairings, and the ligation efficiency in the Ds‐Pa pairing was slightly higher than that in the Ds‐T pairing. In contrast, the L23A strand was efficiently ligated with R23 in both the A‐Pa and A‐T pairings, and the ligation also occurred in the A‐C pairs to some extent.

T4 DNA ligase was more tolerant of base mismatches involving UB and natural base pairings at the second position from the 3′‐termini in the acceptor strands, relative to those at the 3′‐terminal position (Figure 5B). When using the natural‐base acceptor strand, L24AT, all of the cognate and noncognate pairings exhibited high ligation efficiency. In contrast, the Ds pairing with the purine bases of the template strands reduced the ligation efficiency, relative to those in the Ds‐Pa, Ds‐T, and Ds‐C pairings.

## 4 CONCLUSIONS

We have demonstrated ligation reactions involving the hydrophobic Ds‐Pa pair, which functions in replication. Despite the nonhydrogen‐bonded nature of the UBP, T4 DNA ligase efficiently recognizes the Ds‐Pa pairing at the ligation junction with higher selectivity, as compared to that of the A‐T pairing. The Ds base efficiently excludes the noncognate base pairing with the natural bases in ligation. In addition, the ligation efficiency of the Ds‐Pa pair is comparable to that of the A‐T pair. The results indicate that the hydrogen‐bond interactions are less important in enzymatic ligation, as also found in replication. These results encourage us to use the hydrophobic UBPs at ligation junctions in T4 DNA ligation, which could increase the accuracy of the multiple ligations of many DNA fragments. The ligated dsDNA fragments containing the Ds‐Pa pair can be amplified by PCR in the presence of the triphosphate substrates of Ds and Px, as the Ds‐Px pair exhibits high fidelity in PCR. Thus, the combination of the Ds‐Pa pair for ligation and the Ds‐Px pair for PCR enables their practical use in recombinant DNA technology with genetic alphabet expansion. The Ds‐modified Pa pair could be used for DNA assembly with functional residues by ligation.^[^ ^15^, ^37^ ^]^

## CONFLICT OF INTEREST

The authors declare no conflicts of interest.

## Supporting information


**Appendix** **S1:** Supplementary Information

## Data Availability

The data that support the findings of this study are available from the corresponding author upon reasonable request.
