# Supplementary material for: Cognate base‐pair selectivity of hydrophobic unnatural bases in DNA ligation by T4 DNA ligase
Source: Biopolymers. 2020 Nov 6;112(1):e23407. doi: 10.1002/bip.23407 (PMC7900958; doi:10.1002/bip.23407)
Supplement: Supplementary file 1 — Appendix S1: Supplementary Information [file BIP-112-e23407-s001.pdf]

Supporting Information

Cognate base-pair selectivity of hydrophobic unnatural bases in DNA ligation by T4 DNA ligase

Michiko Kimoto[†], Si Hui Gabriella Soh[†,‡], Hui Pen Tan[†], Itaru Okamoto[†], and Ichiro Hirao[†]

[†]Institute of Bioengineering and Nanotechnology, A*STAR, 31 Biopolis Way, The Nanos #07-01, Singapore 138669, [‡]Raffles Institution, 1 Raffles Institution Lane, Singapore 575954

Correspondence should be addressed to M.K. (michiko@ibn.a-star.edu.sg)

CONTENTS

- **Figure S1.** Natural and unnatural base pair structures in DNA duplexes.
- **Scheme S1.** Synthesis of benzoyl-protected Pa phosphoramidite.
- **Figure S2.** ^{1}H NMR spectrum (400 MHz, DMSO-d_6) of 1-(5-O-DMTr-2-deoxy-β-D-ribofuranosyl)-(S)-4-(4,5-dibenzoyloxy-pent-1-yn-1-yl)-1H-pyrrole-2-carbaldehyde phosphoramidite (5).
- **Figure S3.** ^{31}P NMR spectrum (162 MHz, DMSO-d_6) of 1-(5-O-DMTr-2-deoxy-β-D-ribofuranosyl)-(S)-4-(4,5-dibenzoyloxy-pent-1-yn-1-yl)-1H-pyrrole-2-carbaldehyde phosphoramidite (5).
- **Figure S4.** Stability of the 2′-deoxyribonucleoside of Pa under basic conditions.
- **Figure S5.** ESI-MS of d(TPaT).
- **Figure S6.** Enzymatic preparation of Ds-containing DNA fragments, L23Ds and L24DsT.
- **Figure S7.** Ligation of 5′-phosphorylated R18X (donor strand) to L22 (acceptor strand) in the presence of Template 25 (template strand).
- **Figure S8.** Scatter plots of yield [Y(%), the percentage of the ligated 40-mer products / (the ligated 40-mer and non-ligated acceptor strand)] against time (min).
- **Figure S9.** Ligation of 5′-phosphorylated R57 (donor strand) to L23X (acceptor strand) in the presence of Template 25 (template strand).
- **Figure S10.** Scatter plots of yield [Y(%), the percentage of the ligated 40-mer products / (the ligated 40-mer and non-ligated acceptor strand)] against time (min).
- **Table S1.** Sequences of the DNA fragments used in this study.

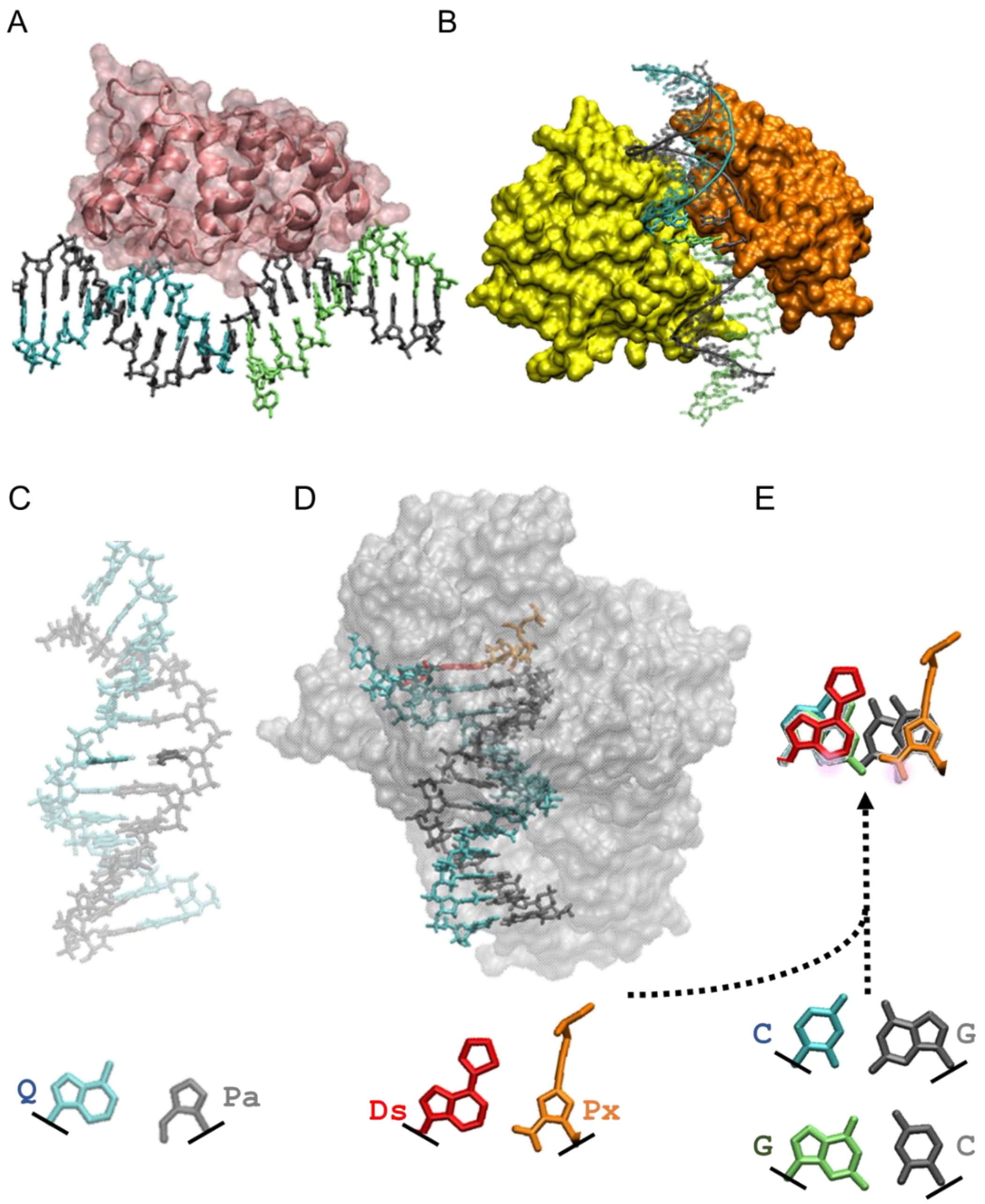

Figure S1. Natural and unnatural base pair structures in DNA duplexes. (A, B) The DNA duplexes, containing the adenylated DNA intermediate, were adopted from PDB: 6D1. (See Figure 1A.) For clear views of the T4 DNA ligase interaction with DNA in Figure 1A, only the NTD (A) or the NTase−OBD (B) within the ligase is shown. (C) The DNA duplex structure containing the Q−Pa pair, determined by NMR analysis (Mitsui *et al.*, *J. Am. Chem. Soc.* 2003, 125, 5298-5307). (D) The Ds–Px pair in a ternary complex of DNA polymerase, Ds-template and primer duplex, and the substrate of Px, determined by X-ray crystallography, adopted from PDB: 5NKL. (E) The base pairs at the nick site in (A, B) were superimposed with the Q–Pa pair in (C) and the Ds–Px pair in (D).

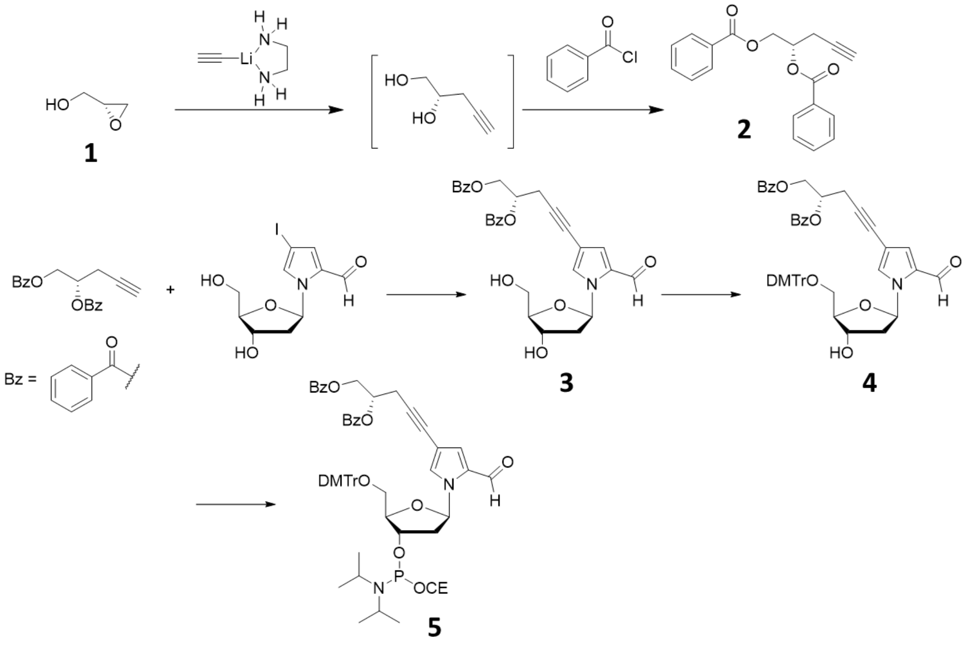

Scheme S1. Synthesis of benzoyl-protected Pa phosphoramidite.

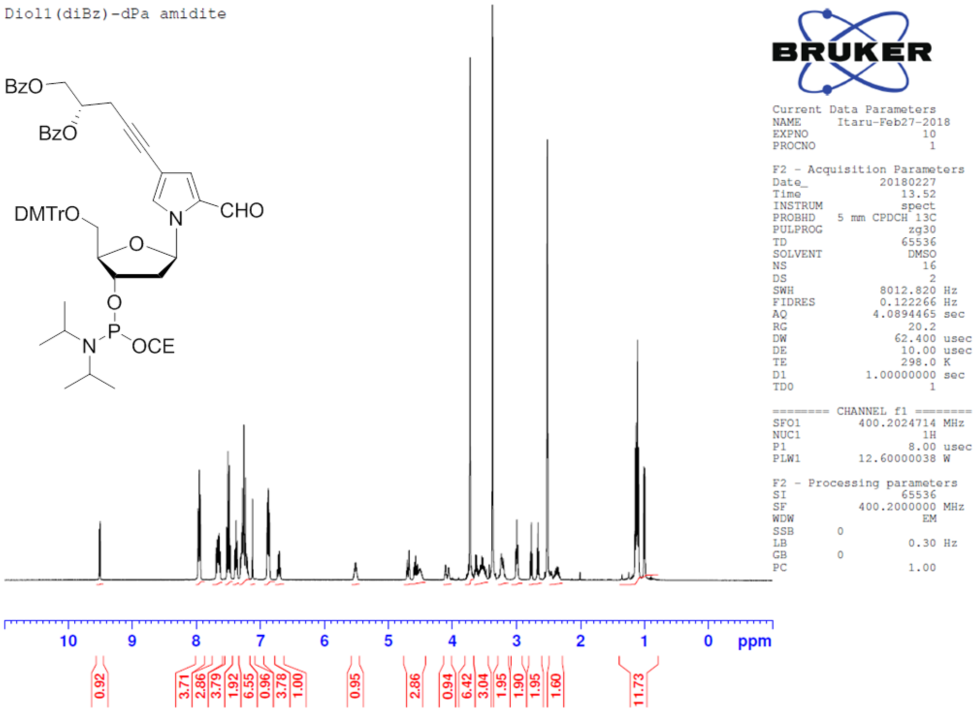

Figure S2. ^{1}**H NMR spectrum (400 MHz, DMSO-d$_6$) of 1-(5-O-DMTr-2-deoxy-β-D-ribofuranosyl)-(S)-4-(4,5-dibenzoyloxy-pent-1-yn-1-yl)-1H-pyrrole-2-carbaldehyde phosphoramidite (5).**

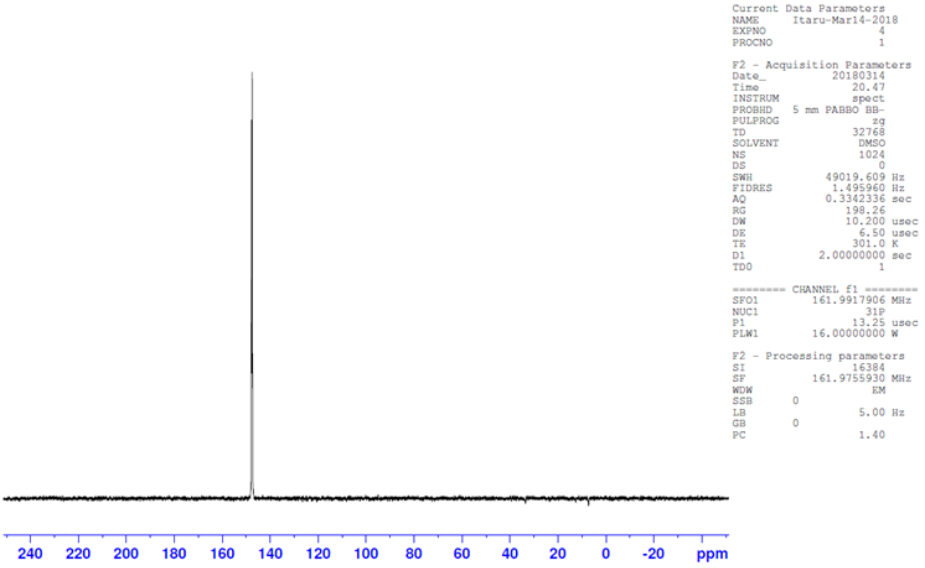

Figure S3. ^{31}P NMR spectrum (162 MHz, DMSO-d6) of 1-(5-O-DMTr-2-deoxy-β-D-ribofuranosyl)-(S)-4-(4,5-dibenzoyloxy-pent-1-yn-1-yl)-1H-pyrrole-2-carbaldehyde phosphoramidite (5).

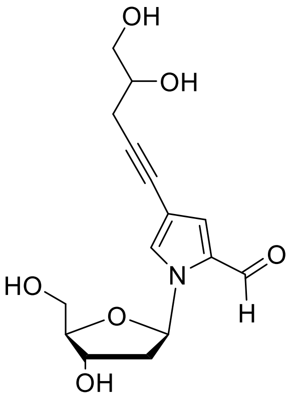

2′-deoxyribonucleoside of Pa (dPa)

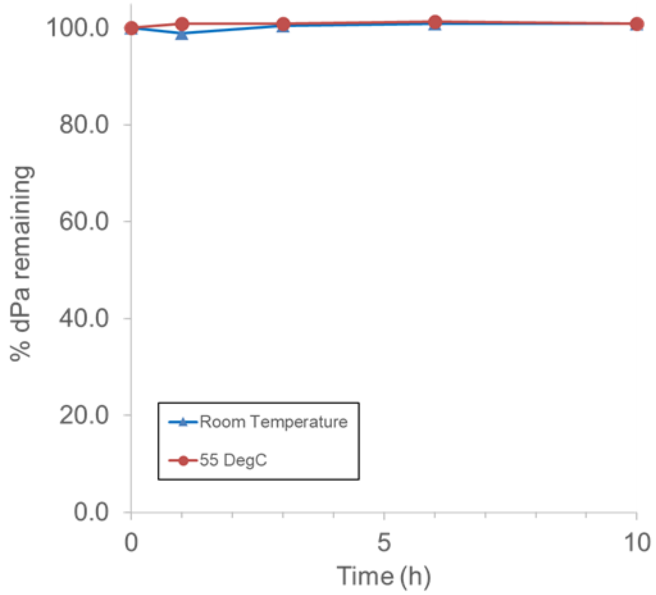

Figure S4. Stability of the 2′-deoxyribonucleoside of Pa under basic conditions. A mixture of dPa (10 mM) and thymidine (10 mM), added as an internal standard, in acetonitrile (ACN): water (20% v/v) (50 µl) was treated with 28% NH_4OH (2.0 ml) at room temperature or heated at 55ºC for 1, 3, 6 or 10 hours in a sample vial (Wheaton sample vial, 4-ml size, with a white rubber-lined cap). The treated solutions were evaporated *in vacuo*, and the residues were dissolved in water. The solutions were analyzed by RP-HPLC (Gilson HPLC system, Capcell PAK C18 column, Shiseido, 250 mm × 4.6 mm ID) under a 10−30% ACN gradient in 100 mM TEAA at a flow rate of 1 ml/min, with detection at 260 nm. The amounts (%) of dPa remaining intact were calculated by normalization to the thymidine peak area.

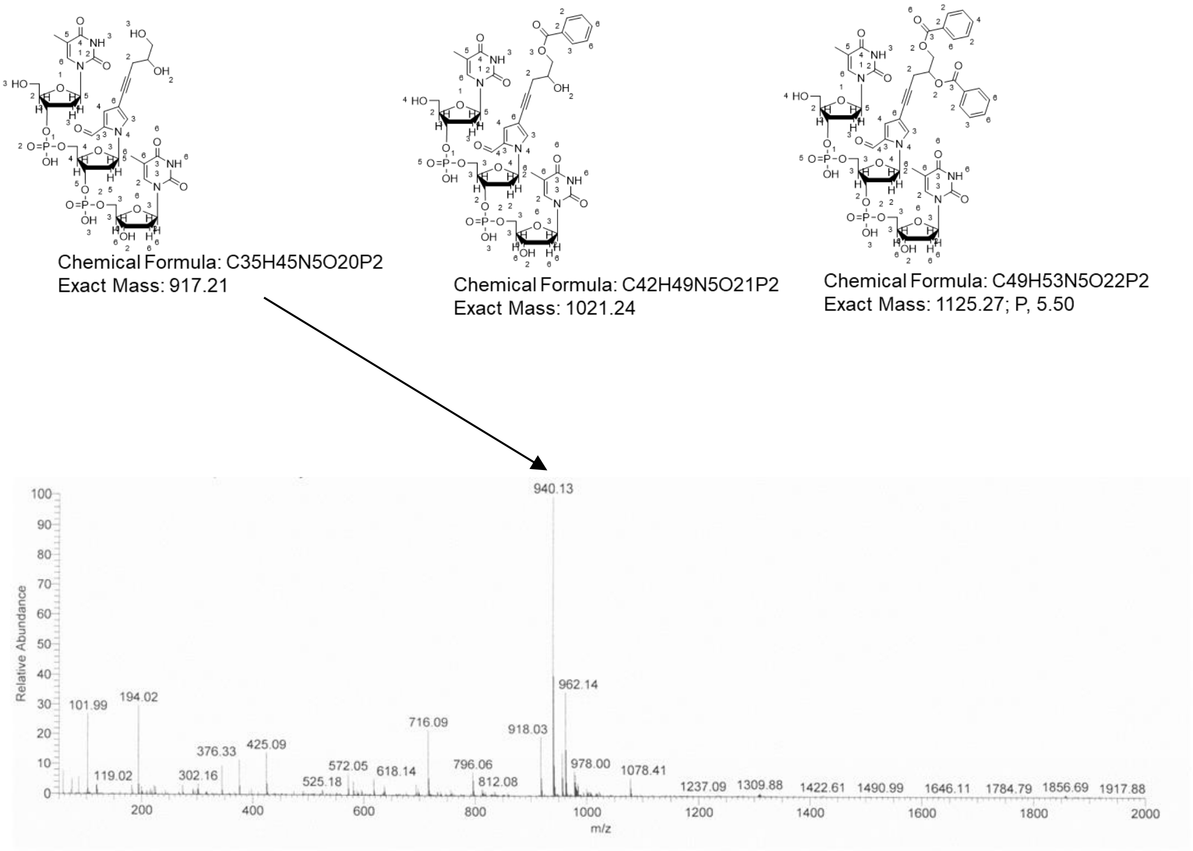

Figure S5. ESI-MS of d(TPaT). The chemically-synthesized trimer oligo, d(TPaT), was purified by reverse-phase HPLC after deprotection with concentrated ammonia solution for one hour at room temperature, for cleavage from the CPG column, and then for six hours at 55°C to remove the two benzoyl groups protecting the diol moiety. ESI-MS was measured with a Thermo Fisher LCQ Fleet Ion Trap Mass Spectrometer. The mass peak for the fully-deprotected oligo was detected in a positive mode as one sodium ion adduct. ESI-MS for $C_{35}H_{45}N_5O_{20}P_2$: calcd. 917.21, found 940.13 $[M+Na]^+$.

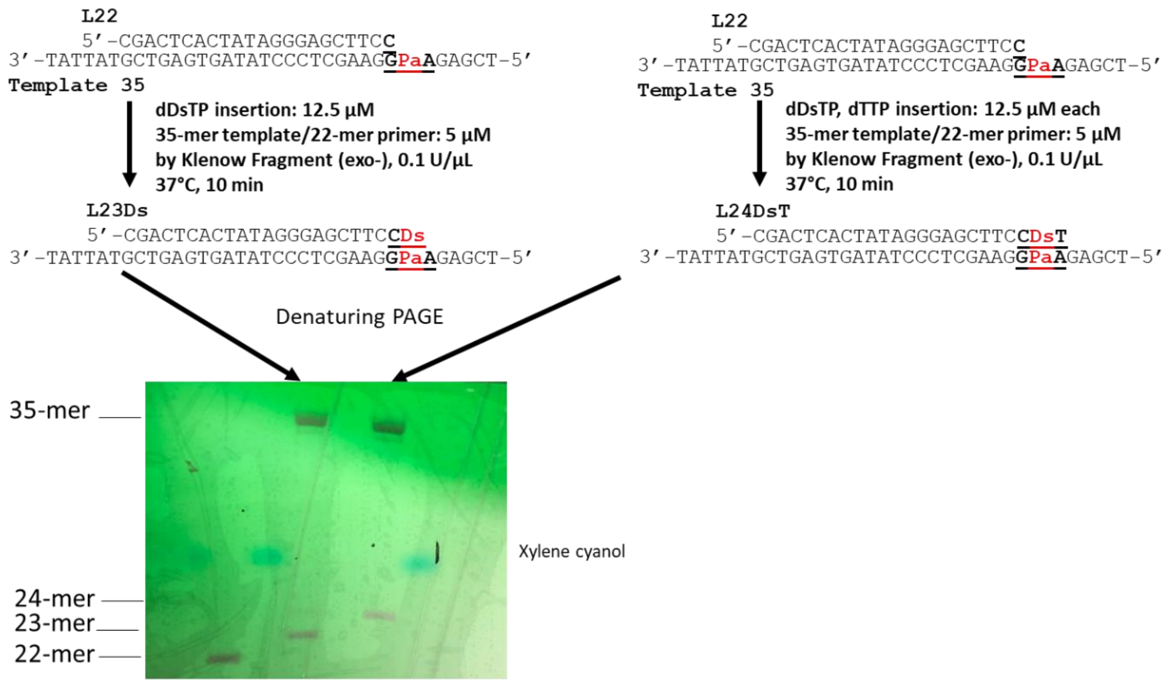

Figure S6. Enzymatic preparation of Ds-containing DNA fragments, L23Ds and L24DsT. A 22-mer primer DNA (L22, 10 µM) was annealed to a 35-mer template containing Pa (Template 35), in annealing buffer (50 mM Tris-HCl, pH 7.0, and 10 mM $MgCl_2$), under the following conditions: 95°C for 30 s, 55°C at 30 s, and 12°C on hold. The annealed DNA solution (80 µl) was mixed with 40 µl of a 50 µM triphosphate solution in reaction buffer (50 mM Tris-HCl, pH 7.5, 10 mM $MgCl_2$, 2 mM DTT and 0.2 mg/ml BSA) and 40 µl of a 0.4 U/µl KF exo- solution in reaction buffer, and then the solution was incubated at 37°C for 10 min. As for the triphosphate solution, only dDsTP was added to the mixture when preparing L23Ds, while only dDsTP and dTTP were added to the mixture when preparing L24DsT. After the reaction was stopped by adding 10 µl of 0.5 M EDTA (pH 8.0), the products were concentrated by ethanol precipitation, and the extended 23-mer and 24-mer products were purified by denaturing PAGE. FAM-L23Ds was also prepared in a similar manner to the non-labeled L23Ds.

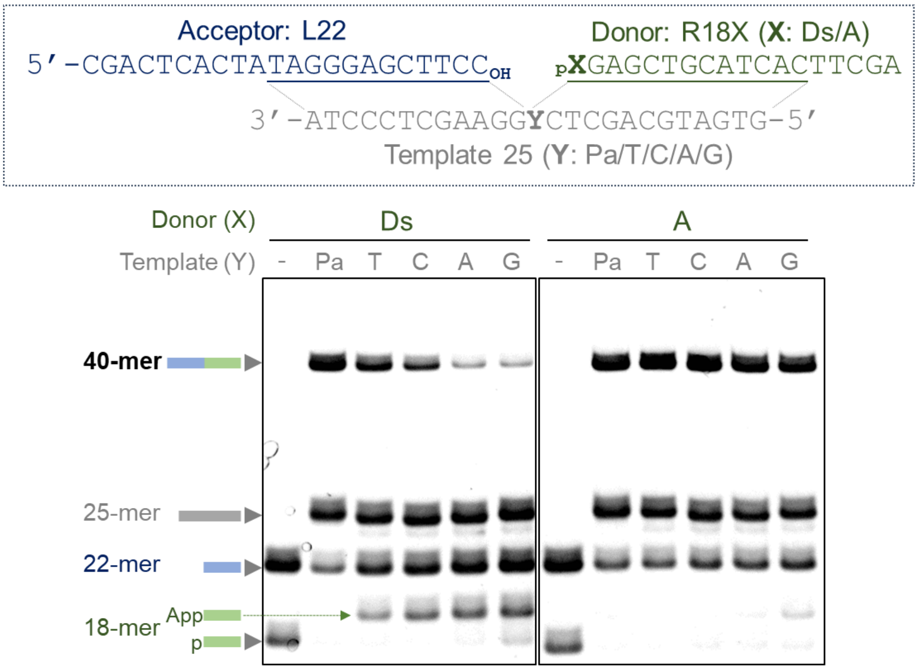

Figure S7. Ligation of 5′-phosphorylated R18X (donor strand) to L22 (acceptor strand) in the presence of Template 25 (template strand). Reaction conditions: 0.25 Weiss U/μL T4 DNA ligase, 0.5 μM each DNA, 10 min at 22°C. X = Ds or A, Y= Pa, T, C, A, or G. The DNA bands in the gel were stained with SYBR Gold and detected with an LAS4000 bio-imager. The ligated products are 40-mers. The 18-mer adenylated donor intermediates are indicated as App.

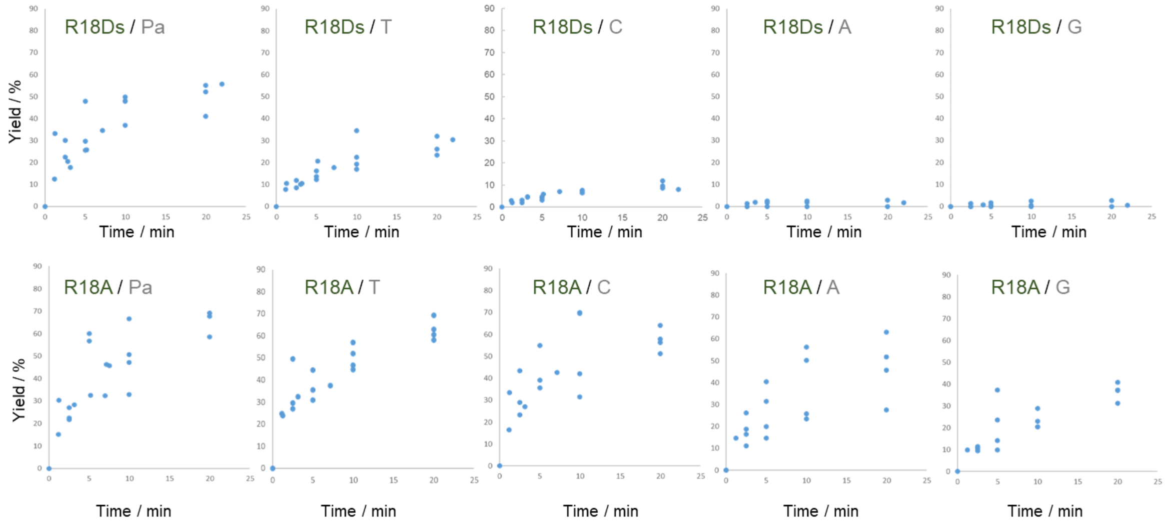

Figure S8. Scatter plots of yield [Y(%), the percentage of the ligated 40-mer products / (the ligated 40-mer and non-ligated acceptor strand)] against time (min). Ligation was performed for R18X (donor strand) in the presence of Template 25. X = Ds or A and Y = Pa, T, C, A, or G. Reaction conditions: 0.0025 Weiss U/μL T4 DNA ligase, 0.5 μM of each DNA fragment.

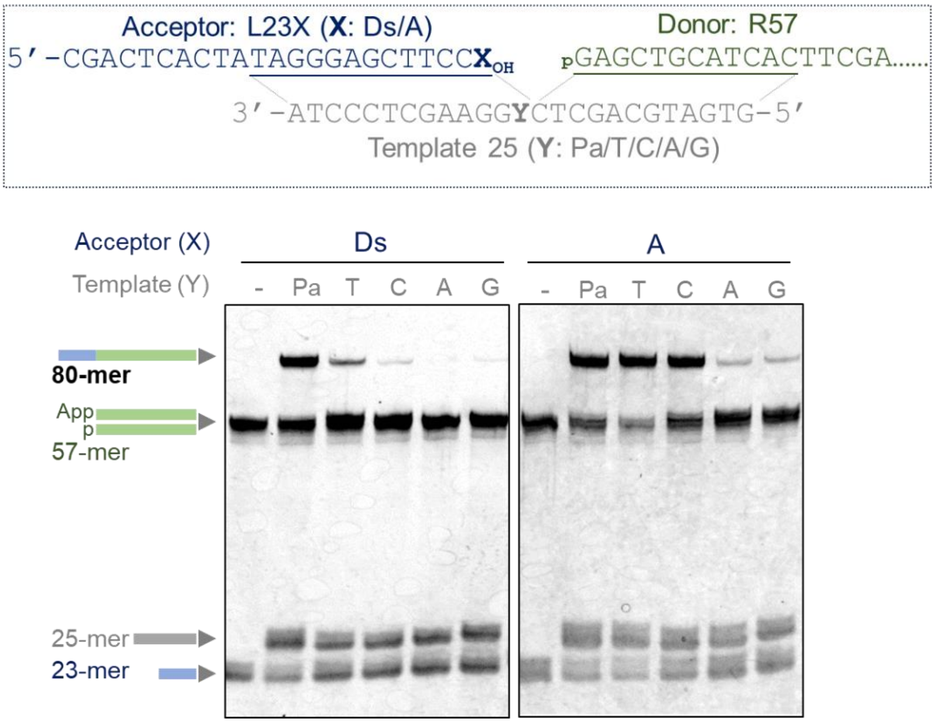

Figure S9. Ligation of 5′-phosphorylated R57 (donor strand) to L23X (acceptor strand) in the presence of Template 25 (template strand). Reaction conditions: 0.25 Weiss U/µL T4 DNA ligase, 0.5 µM of each DNA fragment, 10 min at 22°C. X = Ds or A, Y= Pa, T, C, A, or G.

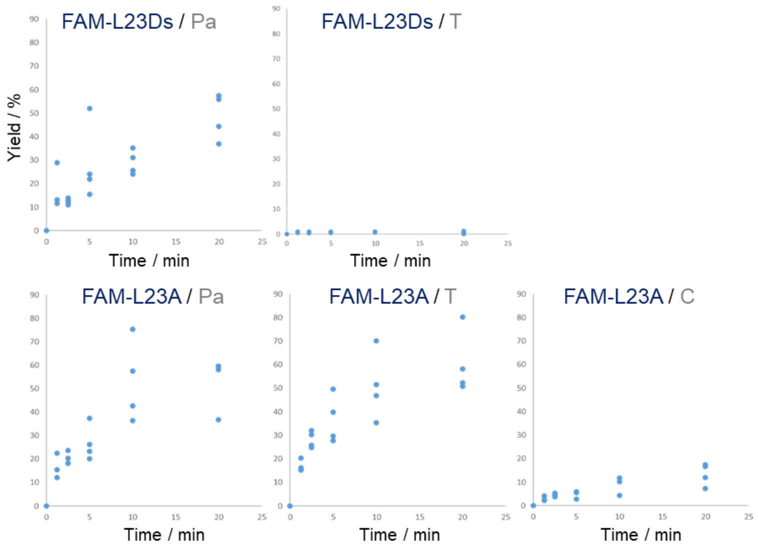

Figure S10. Scatter plots of yield [Y(%), the percentage of the ligated 40-mer products / (the ligated 40-mer and non-ligated acceptor strand)] against time (min). Ligation was performed for R17 (donor strand) and FAM-L23X (acceptor strand) in the presence of Template 25. X = Ds or A and Y = Pa, T, C, A, or G. Reaction conditions: 0.0025 Weiss U/µL T4 DNA ligase, 0.5 µM of each DNA fragment.

Table S1. Sequences of the DNA fragments used in this study. Background colors correspond to the type of use (ligation experiment use as an acceptor strand in blue, as a donor strand in green, and as a template or guide strand in grey).

Name	Sequence (5' to 3', **X** = Ds or A, **Y** = Pa, T, C, A or G)
L22	CGACTCACTATAGGGAGCTTCC
FAM-L22	FAM-CGACTCACTATAGGGAGCTTCC
L23X	CGACTCACTATAGGGAGCTTCC**X**
FAM-L23X	FAM-CGACTCACTATAGGGAGCTTCC**X**
L24XT	CGACTCACTATAGGGAGCTTCC**X**T
R18X	**X**GAGCTGCATCACTTCGA
R17	GAGCTGCATCACTTCGA
R57	GAGCTGCATCACTTCGAGGATGTCTTCGTGTCAGACTACGATAGCCT GGATCCCGCT
R23	TCTCGACTGGATCCCGCTACGCA
R22	CTCGACTGGATCCCGCTACGCA
Template 25	GTGATGCAGCTC**Y**GGAAGCTCCCTA
Template 35	TCGAGA**Y**GGAAGCTCCCTATAGTGAGTCGTATTAT
17-guide	TGCGTAGCGGGATCCAG
